# ATP11A promotes EMT by regulating Numb PRR^L^ in pancreatic cancer cells

**DOI:** 10.7717/peerj.13172

**Published:** 2022-03-23

**Authors:** Lin Chen, Jingtong Tang, Weiwei Sheng, Jian Sun, Yuteng Ma, Ming Dong

**Affiliations:** Department of Gastrointestinal Surgery, The First Hospital of China Medical University, Shenyang, China

**Keywords:** ATP11A, Numb, ZEB1, Snail2, Pancreatic cancer, EMT

## Abstract

**Purpose:**

The Numb protein plays a vital role in tumor development. The main aim of this study was to identify ATP11A, which is associated with the biological behavior of pancreatic cancer, and elucidate its relationship with Numb and the underlying mechanism behind this relationship.

**Methods:**

First, data retrieved from The Cancer Genome Atlas (TCGA) and Genotype-Tissue Expression (GTE_X_) databases was used to investigate the expression of ATP11A mRNA and its relationship with Numb mRNA in pancreatic cancer. Western blot assays on 31 pairs of pancreatic cancer tissues and paracancerous tissues, and immunohistochemical assays on 81 pancreatic cancer specimens were performed in order to verify the expression of ATP11A in pancreatic cancer at the protein level. Next, ATP11A was overexpressed or knocked down to observe its effects on the invasion and migration ability of pancreatic cancer cells and the changes of downstream proteins. Rescue assays were conducted to determine the mechanism through which ATP11A affects Numb, ZEB1, Snail2 and other proteins. Furthermore, immunoprecipitation assays were performed to explore the interaction between ATP11A and Numb. Finally, pancreatic cancer cells were stimulated with TGFB1 and ATP11A expression was examined to explore whether the effect of ATP11A on EMT was TGFB dependent.

**Results:**

At the mRNA level, the expression of ATP11A in pancreatic cancer tissues was significantly higher than in normal pancreatic tissues (*P* < 0.001). ATP11A expression was also highly correlated with Numb expression (R = 0.676). At the protein level, ATP11A expression in pancreatic cancer tissues was significantly higher than that in paracancerous tissues (*P* = 0.0009), and high ATP11A expression was also correlated with a worse prognosis. Moreover, our results showed that ATP11A can promote the invasion and migration of pancreatic cancer cells. Additionally, ATP11A could positively regulate the expression of Numb PRR^L^, Snail2 and ZEB1 proteins. The rescue experiment results showed that the enhancement effect of ATP11A on ZEB1/Snail2 was suppressed by the specific knockdown of Numb PRR^L^. In addition, the immunoprecipitation results showed that ATP11A could specifically bind to Numb PRR^L^. The expression of ATP11A was also upregulated after TGFB stimulation, suggesting that the effect of ATP11A on EMT is TGFB dependent.

**Conclusion:**

ATP11A is significantly upregulated in pancreatic cancer tissues, where it promotes the invasion and migration ability of pancreatic cancer cells. It is also associated with adverse prognosis in pancreatic cancer. Furthermore, ATP11A affects the epithelial-to-mesenchymal transition (EMT) of pancreatic cancer by regulating the TGFB dependent Numb PRR^L^-ZEB1/Snail2 pathway.

## Introduction

The current 5-year overall survival rate of pancreatic cancer (PC) patients is only 6% (McGuigan et al., 2018). In 2018, global cancer statistics (Bray et al., 2018) indicated that new cases of pancreatic cancer accounted for only 2.5% of all new cancer diagnoses, but pancreatic cancer-related deaths accounted for 4.5% of all cancer-related deaths. Cases of pancreatic disease are rapidly rising in the western world, causing major concern (Bray et al., 2018). Saad et al. (2018) reported that from 1973 to 2014, the ASIR (age-standardized incidence) of pancreatic cancer grew by 1.03% annually, implying that pancreatic cancer will have ascended from the fourth leading cause of cancer-related deaths in the US to the second by 2030 (Rahib et al., 2014; Siegel, Miller & Jemal, 2017). This trend highlights the need to identify new targets for pancreatic cancer treatment.

ATP11A, also known as ATPase Phospholipid Transport 11A, is a catalytic component of the P4-ATPase inverting enzyme complex in mammals (Segawa, Kurata & Nagata, 2016). It catalyzes the hydrolysis of ATP coupled with the transport of aminophospholipids, phosphatidylserines (PS) and phosphatidylethanolamines (PE), from the outer to the inner leaflet of the plasma membrane and contributes to the maintenance of membrane lipid asymmetry (Segawa, Kurata & Nagata, 2016). Currently, ATP11A is being studied in several tumors (Geybels et al., 2017; Hu et al., 2019; Miyoshi et al., 2009; Zhao et al., 2017). One study identified ATP11A as a predictive prognosis marker of colorectal cancer (CRC) (Miyoshi et al., 2009). Other studies have identified the methylated ATP11A gene as a prognostic marker for acute myeloid leukemia (Hu et al., 2019) and prostate cancer (Geybels et al., 2017; Zhao et al., 2017).

The Numb protein helps determine cell fate in Drosophila (Uemura et al., 1989). Mammalian Numb is an evolutionarily conserved signaling adapter protein that also determines cell fate (Cayouette & Raff, 2002; Verdi et al., 1996). In addition, Numb plays an important role in some cancers (Pece et al., 2004; Westhoff et al., 2009). For example, it is considered to be a tumor suppressor in pancreatic cancer (Sheng et al., 2017). A previous study reported that the alternative splicing of Numb transcripts leads to two main isoforms that vary in the length of their proline-rich regions (PRR) length (Yan, 2010). The isoform that includes an exon 12-encoded 48–amino acid insert is called PRR^L^. Conversely, the isoform that excludes the exon 12-encoded 48–amino acid insert is called PRR^S^. These two isoforms assume different roles in managing cell functions. Another study reported that Numb PRR^L^ advanced proliferation, but had no impact on differentiation, while Numb PRR^S^ advanced differentiation but did not affect proliferation in murine embryonic carcinoma cells (Verdi et al., 1999). Furthermore, the expression of Numb PRR^L^ was higher in liver cancer tissues compared to normal tissues and the expression of ATP11A was correlated with adverse prognosis (Lu et al., 2015). In this study, the role of ATP11A and its association with Numb PRR^L^ in pancreatic cancer (PAAD) was investigated.

## Material and Methods

### Confirmation statement

We confirm that all experiments were carried out in accordance with the relevant guidelines and regulations.

### Bioinformatic analysis

The data of 178 pancreatic cancer patients were retrieved from the TCGA (The Cancer Genome Atlas) database, and the data of 171 normal pancreas were retrieved from the GTE_X_ (Genotype-Tissue Expression) database. A differential expression analysis was performed between the cancer tissues and the normal issues using Sangerbox tools, a free online platform for data analysis (http://www.sangerbox.com/tool). The relationship between ATP11A and Numb was determined based on the data of 178 pancreatic cancer patients obtained from the TCGA database using the online tool, ENCORI (Li et al., 2014).

### Cell culture and tissue samples

The Ethics Committee of the First Hospital of China Medical University approved this study (Committee Approval Number: AF-SOP-07-1.1-01). Human pancreatic carcinoma cell lines, SW1990 and PANC-1, were purchased from the Chinese Academy of Sciences Cell Bank (Shanghai, China). All cell lines were maintained in RPMI-1640 medium with 10% FBS (Gibco Invitrogen, Carlsbad, CA, USA). Cells were grown at 37 °C in a humidified 5% CO_2_ incubator.

In addition, pancreatic cancer samples were acquired from patients who had surgical resections. It is worth noting that the application for the exemption of informed consent was approved by the Ethics Committee of the First Hospital of China Medical University.

### Cell transfection

For cell transfection, Lipofectamine™ 3000 reagent protocol was used. ATP11A plasmids (Genechem, Shanghai, China), NC plasmids (Genechem, Shanghai, China), ATP11A siRNA (GenePharma, Shanghai, China), Numb PRR^L^ siRNA (GenePharma, Shanghai, China), and negative control oligonucleotides (GenePharma, Shanghai, China) were transfected using the Lipofectamine 3000 (Invitrogen, Waltham, CA, USA). First, seed cells to be 70–90% confluent at transfection. Second, dilute the Lipofectamine 3000 reagent in Opti-MEM medium and prepare the master mix of DNA by diluting the DNA in Opti-MEM medium, then add P3000 reagent. Third, add diluted DNA to the diluted Lipofectamine 3000 Reagent. Then incubate and add DNA-lipid complex to cells. Incubate cells for 48 h and then perform the subsequent experiments. To transfect the cells with siRNA, follow the protocol as described for DNA but do not add P3000 reagent when diluting the siRNA. File S1 contains these sequences.

### Western blot

Pancreatic cancer cells were seeded in 12-well plates (1 × 10^5^ cells/well) and harvested 48 h after transfection. Proteins were extracted from tissues or cells using RIPA Lysis buffer supplemented with phenylmethylsulfonyl fluoride, protease inhibitor, phosphatase inhibitor. Protein concentration was quantified using bicinchoninic acid (BCA) protein concentration determination kit (TaKaRa, Japan). Samples were then resolved on 10% SDS–polyacrylamide gels and transferred to nitrocellulose. PVDF membranes were blocked with 5% skimmed milk for 2 h, then incubated overnight with primary antibodies against: E-cadherin (Abcam, Cambridge, UK), N-cadherin (Proteintech, Rosemont, IL, USA), Fibronectin (Proteintech, Rosemont, IL, USA), Vimentin (Proteintech, Rosemont, IL, USA), Snail2 (Proteintech, Rosemont, IL, USA), ZEB1 (Proteintech, Rosemont, IL, USA), Numb (Abcam, Cambridge, UK), ATP11A (Abcam, Cambridge, UK), SRPK2 (Abcam, Cambridge, UK) and GAPDH (Proteintech, Rosemont, IL, USA). The second day, secondary antibodies incubations were performed using horseradish peroxidase(HRP)-conjugated secondary antibodies (Proteintech, Rosemont, IL, USA). Finally, an ECL-chemiluminescent kit (Thermol Biotech Inc, Waltham, MA, USA) was used to detect the protein bands.

### Immunohistochemistry (IHC)

Paraffin-embedded tissues were dewaxing with dimethylbenzene and treated with ethanol at various concentrations. Antigen retrieval was performed using the high pressure method. A total of 3% hydrogen peroxidase was used to quench the endogenous peroxidase activity. The tissues were subsequently incubated with 10% ordinary goat serum. Next, sections were incubated overnight with anti-ATP11A primary antibodies (Abcam, Cambridge, UK, 1:200). Then secondary antibodies were added to incubate the sections. Diaminobenzidine (DAB) was utilized to visualize the sections, followed by counterstainining with hematoxylin. Sections were then observed and positive cells counted under a microscope. For each section, five high-power fields (magnification × 400) were randomly chosen. Staining intensity was scored 0 (negative) to 3 (strong intensity staining). Based on the percentage of staining, the extent was scored as 0 (<5%), 1 (5–25%), 2 (26–50%), 3 (51–75%), or 4 (>75%). IHC was evaluated by three independent pathologists. These two scores were then multiplied to make a final IHC score ranging from 0 to 12. Positive expression was defined as a IHC score >8, due to the high expression of ATP11A in pancreatic cancer tissues. Setting the standard as eight allowed us to better distinguish the clinicopathological parameters of the high expression group from the low expression group.

### Transwell invasion and migration assay

Transfected cells were collected 48 h later after transfection and then resuspended in the serum-free RPMI-1640 culture medium. A total of 3 × 10^4^ transfected cells were placed on the top compartment pretreated with Matrigel. The lower chamber was filled with medium containing 10% serum. Cold methyl alcohol was used to fix the cells on the lower membrane. Then cells were stained using 1% crystal violet stain solution (Solarbio, Beijing, China). Finally, invaded cells were counted in five arbitrarily chosen fields for each chamber. Fields were counted by different authors that were blind to conditions. The protocol for the cell migration assay was similar to the invasion assay protocol, with the exception of Matrigel coating.

### RNA extraction and rt-PCR validation

RNA from tissue samples was extracted using TRIzol reagent (TAKARA, Japan). Complementary DNA (cDNA) was synthesized using the PrimeScript RT reagent Kit with gDNA Eraser (TAKARA, Japan). qPCR was performed with a TB Green Premix Ex Taq II (TAKARA, Japan) with a QuantStudio 3 Real-Time PCR Instrument (Applied Biosystems, Waltham, MA, USA). The sequences of PCR primers of Numb PRR^L^ are CTTCCAAGCTAATGGCACTG (Forward primer) and CTCTTAGACACCTCTTCTAACCA (Reverse primer). The PCR cycling conditions were as follows: 95 °C for 30 s, 45 cycles of 95 °C for 5 s, 70 °C for 45 s, dissociation at 95 °C for 15 s, 60 °C for 1 min and 95 °C for 1 s. The results were analyzed using the 2(−ΔΔC(T)) Method.

### Immunoprecipitation

For immunoprecipitation, Santa Cruz Biotechnologys Immunoprecipitation protocol was used. Entire protein lysates were extracted using RIPA lysis buffer supplemented with 1 mM phenylmethylsulfonyl fluoride (PMSF), protease inhibitor, phosphatase inhibitor (Bimake, Houston, TX, USA). Lysates were precleared by adding a 1:2 protein A/G PLUS agarose bead slurry (Santa Cruz Biotechnology, Santa Cruz, CA, USA), incubating for 10 min with agitation, centrifuging, and retaining the resulting supernatant. The rabbit polyclonal antibody ATP11A (Abcam, Cambridge, UK) and mouse monoclonal antibody IgG (Santa Cruz Biotechnology, Santa Cruz, CA, USA) antibodies were preincubated with magnetic beads. Next, the antibody-beads complex was washed three times with lysis buffer and incubated with the soluble supernatants of the protein lysates overnight at 4 °C. After centrifuging the samples at 14,000 rpm, the pellet was washed with PBS, resuspended, and boiled for 5 min to remove immunocomplexes from the beads before centrifugation to pellet the beads. Subsequently, the material was eluted and performed a SDS-PAGE test and western blot analysis.

### EMT construction

TGFβ1 (10 ng/mL) (Peprotech, RockyHill, NJ, USA) was applied to PANC-1 and SW1990 cells for 48 h. Both cell lines received a control treatment of 1% bovine serum albumin (Sigma-Aldrich, St. Louis, MO, USA). Cells were cultured in growth media containing 1% fetal calf serum, as recommended. EMT was discovered by observing EMT-like cell morphology and invasion, as well as changes in E-cad, Vimentin, and N-cad protein levels.

### Statistical analysis

All data are presented as the means ± SEM of at least three independent experiments. Differences between two groups were assessed using Student’s *t*-test, while differences between multiple groups were determined by one-way ANOVA. The correlation of Numb and ATP11A was analyzed using the Pearson correlation coefficient. The clinicopathologic significance of ATP11A expression was analyzed using the Chi-squared test. The overall survival was analyzed using Kaplan–Meier curves, while the log-rank test was used for comparison. The differences of WB analysis, cell migration and invasion assays were compared using Student’s *t*-test. SPSS software (SPSS 21.0; SPSS Inc., Chicago, IL, USA) was used for statistical testing, and a *P-*value of less than 0.05 was considered to be significant.

## Results

### A bioinformatics analysis found that ATP11A was highly expressed in pancreatic cancer and had a strong correlation with Numb

**Figure 1:**
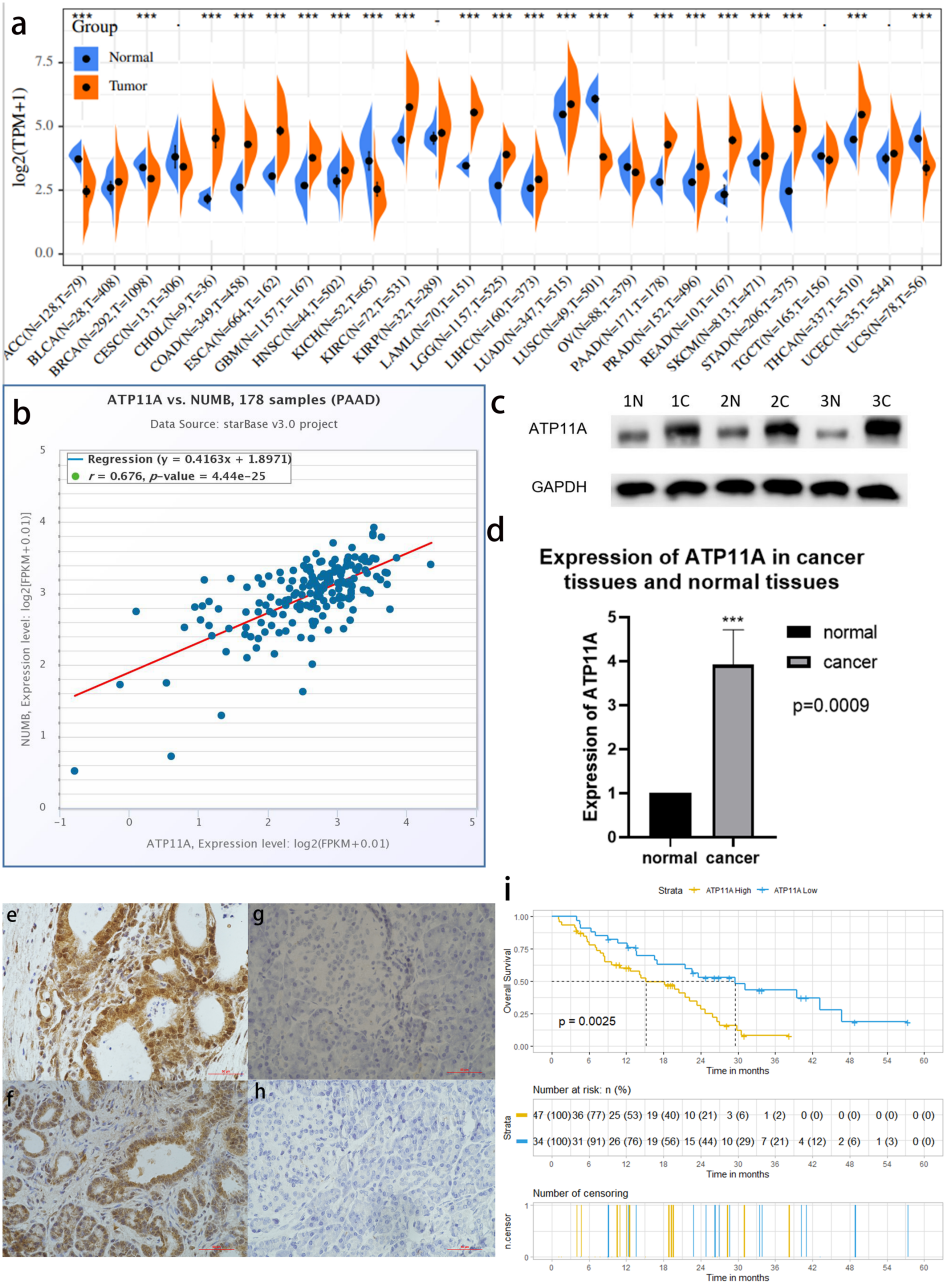
ATP11A is highly expressed in pancreatic cancer tissues and is associated with poor prognosis. (A) A comparison of 178 TCGA pancreatic cancer tissues and 171 GTE_X-_normal pancreatic tissues indicated that ATP11A mRNA expression was significantly higher in pancreatic cancer tissues than in normal pancreatic tissues (*P* < 0.001). (B) The expression of ATP11A mRNA and Numb mRNA was strongly correlated in 178 pancreatic cancer cases in the TCGA database (Pearson correlation coefficient R = 0.676). (C, D) The expression of the ATP11A protein in pancreatic cancer tissues and paracancerous tissues. (C) Western blot analysis showed that ATP11A expression was significantly higher in pancreatic cancer tissues than in paracancerous tissues. (D) ATP11A protein expression was significantly higher in pancreatic cancer tissues than in paracancerous tissues according to the ratio of ATP11A expression to the corresponding paracancerous tissues in 31 pairs (*P* = 0.009). The differences of the WB analysis were expressed as mean ± SEM and compared using a ratio paired *t*-test. (E–H) Immunohistochemical results showed that the expression of ATP11A was significantly higher in pancreatic cancer tissues (E, F) than in paracancerous tissues (G, H). (i) 81 pancreatic cancer tissues were divided into two groups according to immunohistochemical scores, and the group with high ATP11A expression had a worse overall survival rate. A Kaplan–Meier curve was used to estimate survival. *P < 0.05; ***P < 0.001.

Among 27 types of malignant tumors, ATP11A was highly expressed in 16 types of tumors, had a low expression in six types of tumors, and had no statistically significant difference in five types of tumors, indicating that ATP11A is generally overexpressed in tumors. In pancreatic cancer tissues, ATP11A expression was remarkably higher than in normal tissues (*P* < 0.001; Fig.1A). In addition, Numb was strongly correlated with the expression of ATP11A (R = 0.676; Fig.1B).

### A western blot analysis verified the high expression of ATP11A in pancreatic cancer

The expression of ATP11A protein in 31 pancreatic cancer tissues and corresponding paracancerous tissues was measured using western blot. (Fig.1C). The results showed that the expression of ATP11A was significantly higher in pancreatic cancer tissues than in paracancerous tissues (*P* = 0.0009; Fig.1D).

### Immunohistochemistry confirmed that ATP11A expression was enhanced in pancreatic cancer tissues and correlated with clinicopathological parameters

The immunohistochemical results of 81 pancreatic cancer cases showed that ATP11A was mainly expressed in the ductlike structure of pancreatic cancer, and was expressed in the cytomembrane and cytoplasm of pancreatic cancer cells. The expression of ATP11A was higher in cancer tissues (Figs. 1E, 1F ) than in paracancerous tissues (Figs. 1G, 1H), and was associated with the AJCC stage of pancreatic cancer (*P* = 0.045; Table 1). Moreover, the low ATP11A expression group had a better prognosis than the high ATP11A expression group (*P* = 0.0025) (Fig. 1I).

**Table 1:** Correlations between ATP11A and clinicopathologic parameters in PC patients.

Parameters	ATP11A expression		X^2^	*P* value
	Low	High		
No.	34	47		
Gender			0.591	0.442
Male	21	25		
Female	13	22		
Age			0.058	0.810
>65	8	10		
≤65	26	37		
Tumor location			0.100	0.752
pancreatic head	25	36		
pancreatic body and tail	9	11		
Differentiation			0.034	0.983
Well	10	13		
moderate	22	31		
Poor	2	3		
T stage			2.166	0.339
T1	5	9		
T2	27	31		
T3	2	7		
Lymph node status			3.142	0.076
Negative	29	32		
Positive	5	15		
AJCC stage			4.035	0.045
I+IIA	28	29		
IIB+III	6	18		

### ATP11A could induce EMT and regulate the expression of Numb PRR^L^ and EMT-related proteins

**Figure 2:**
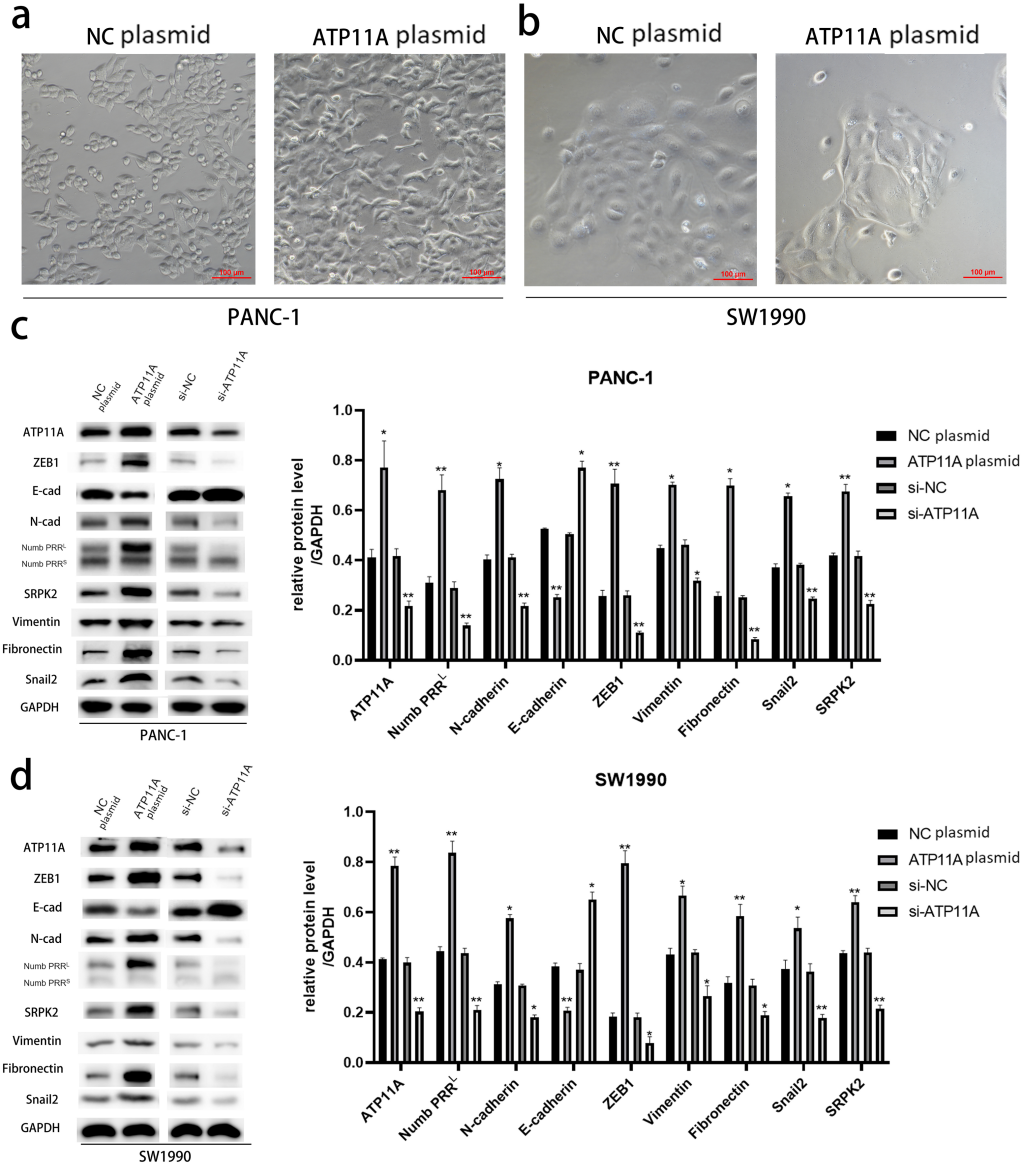
*ATP11A* promotes EMT of PANC-1 and SW1990 cells. (A, B) EMT-like changes were observed in the cell morphology of PANC-1 (A) and SW1990 (B) 48 h after transfection with ATP11A overexpressed plasmid. (C, D) ATP11A regulates the expression of Numb PRR^L^ and EMT-related proteins. (C) Changes in related downstream proteins after ATP11A over-expression or knockdown in PANC-1 cells. (D) Changes in related downstream proteins after ATP11A over-expression or knockdown in SW1990 cell. Each experiment was repeated three times. The differences of WB analysis were expressed as mean ± SEM and compared by Student’s *t*-test. *P < 0.05; **P < 0.01.

After transfection of PANC-1 and SW1990 cells with ATP11A overexpression plasmid, significant changes were found in cell morphology (Figs. 2A, 2B ), which indicated that the overexpression of ATP11A promoted the occurrence of EMT in pancreatic cancer cells. WB experiments were conducted to verify our conjecture. According to the western blot analysis results, the overexpression of ATP11A led to a concomitant increase in the expression of Numb PRR^L^, ZEB1, Fibronectin, N-cadherin, Vimentin, Snail2 and SRPK2, and decreased the expression of E-cadherin. On the other hand, ATP11A knockdown decreased the expression of Numb PRR^L^, ZEB1, Fibronectin, N-cadherin, Vimentin, Snail2 and SRPK2 and increased the expression of E-cadherin (Figs. 2C, 2D).

### ATP11A could significantly promote the invasion and migration ability of pancreatic adenocarcinoma cells

**Figure 3:**
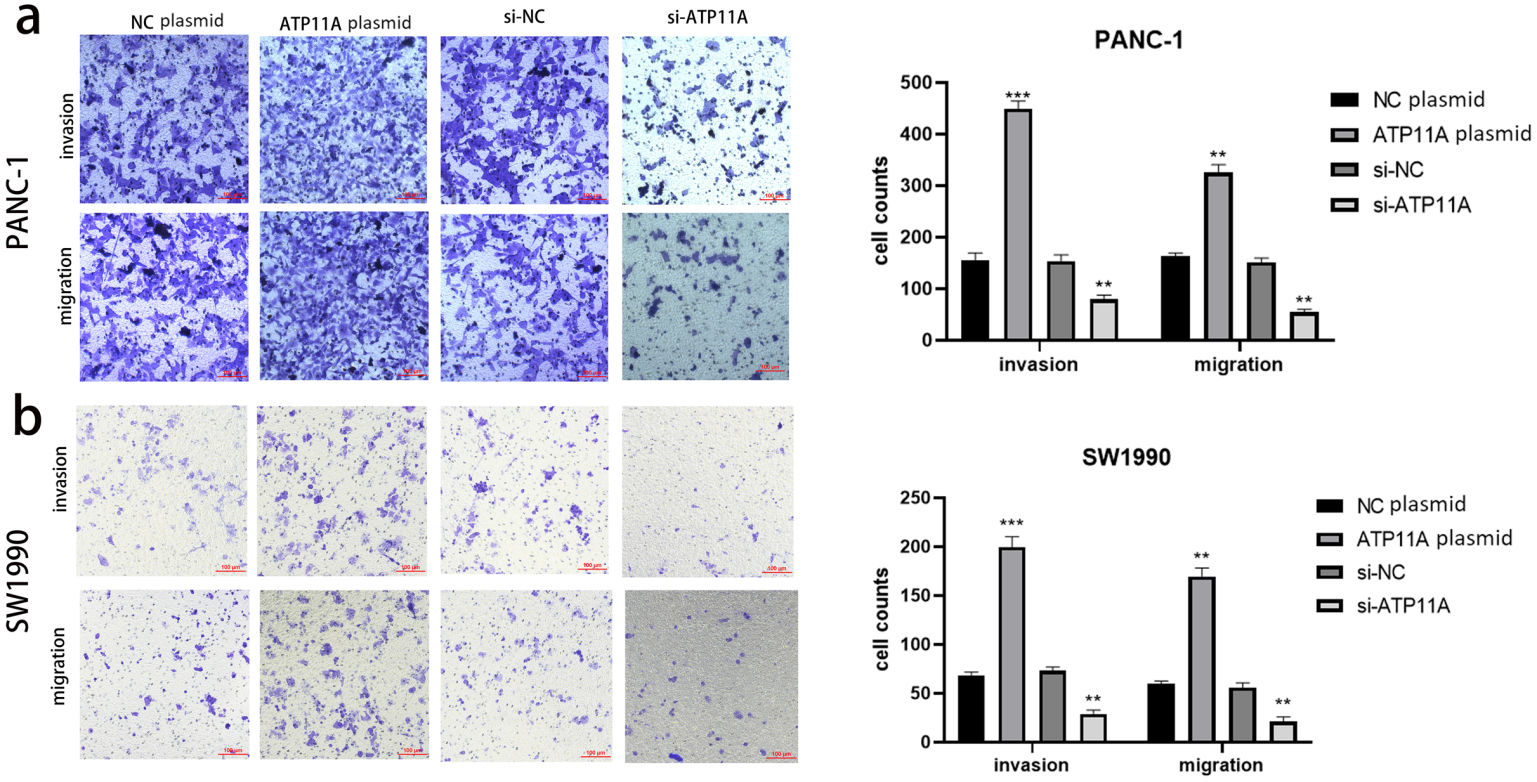
ATP11A affects the invasion and migration ability of pancreatic cancer cells. (A) The invasion and migration ability of PANC-1 cells was increased when ATP11A was over-expressed, and decreased when ATP11A was knocked down. (B) The invasion and migration ability of SW1990 cells was increased when ATP11A was over-expressed, and decreased when ATP11A was knocked down. Each experiment was repeated three times. The differences of cell migration and invasion assays were expressed as mean ± SEM and compared by the Student’s *t*-test. **P < 0.01; ***P < 0.001.

Cell function experiments were conducted to further explore the effects of ATP11A on the biological behavior of pancreatic cancer cells. The invasion and migration experiments showed that increased ATP11A expression significantly promoted the invasion and migration ability of pancreatic adenocarcinoma cells, while decreased ATP11A expression significantly decreased the invasion and migration ability of pancreatic adenocarcinoma cells (Figs. 3A, 3B).

### Numb PRR^L^ is highly expressed in pancreatic cancer tissues, and is important for maintaining the aggressive pancreatic cancer cell phenotype and EMT

**Figure 4:**
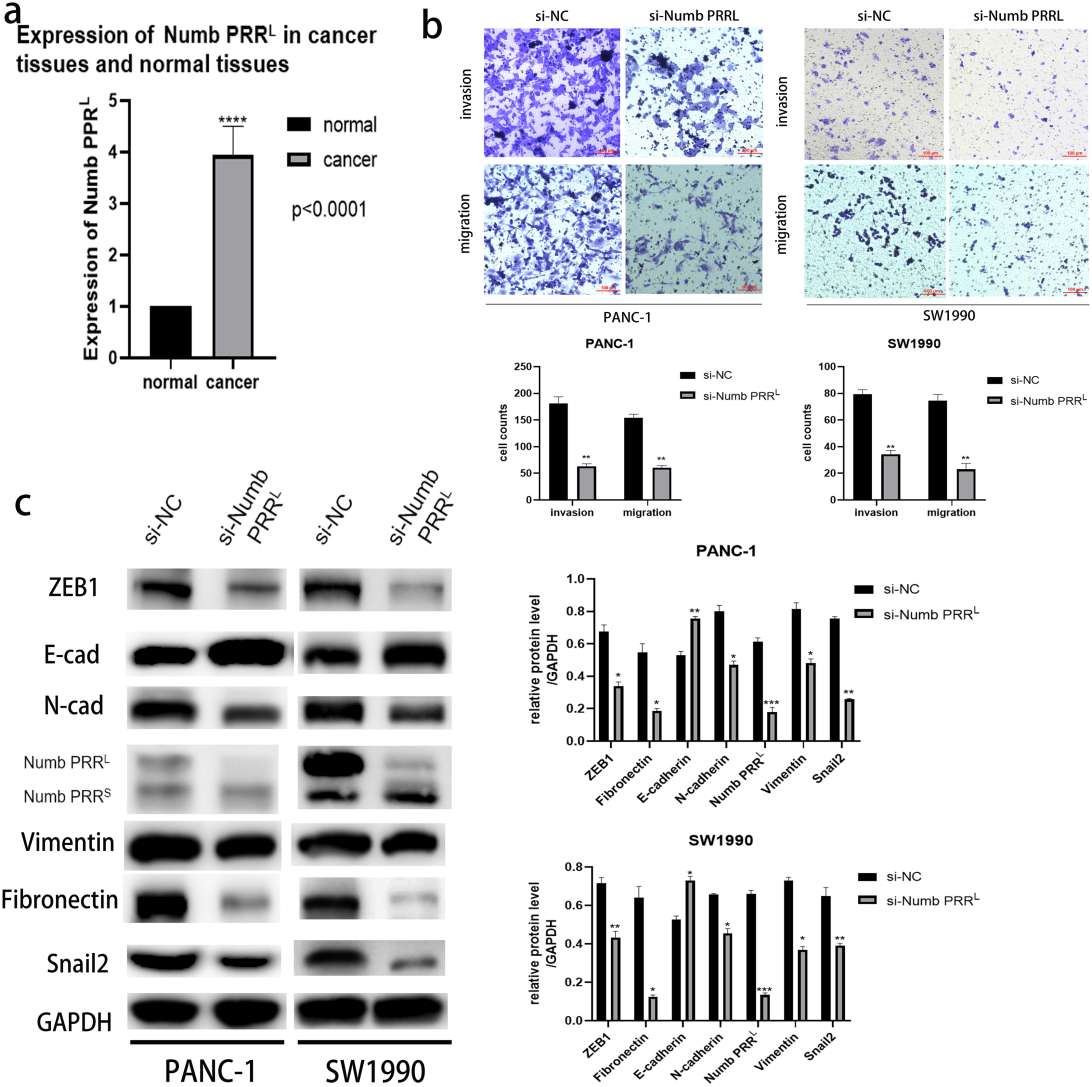
Numb PRR^L^ is important for maintaining the aggressive pancreatic cancer cell phenotype and EMT. (A) Numb PRR^L^ is highly expressed in pancreatic cancer tissues. The results were analyzed using the 2(−ΔΔC(T)) Method. The differences of rt-PCR analysis were expressed as mean ± SEM and compared by a ratio paired *t*-test. (B) Numb PRR^L^ could significantly promote invasion and migration abilities of PANC-1 and SW1990 cells. (C) Numb PRR^L^ regulates the expression EMT-related proteins. Related downstream proteins were changed after Numb PRR^L^ knock down in PANC-1 and SW1990 cells. Each experiment was repeated three times. The differences of WB analysis, cell migration and invasion assays were expressed as mean ± S.E. and compared by the Student’s *t*-test. *P < 0.05; **P < 0.01; ***P < 0.001; ****P < 0.0001.

After confirming the association between ATP11A and Numb PRR^L^ and the promoting effect of ATP11A on EMT, we decided to explore the expression of Numb PRR^L^ in pancreatic cancer tissues and whether Numb PRR^L^ affects EMT. Rt-PCR results showed that Numb PRR^L^ expression in pancreatic cancer tissues was higher than that in paracancer tissues (Fig. 4A). In order to further investigate the effects of Numb PRR^L^ on the invasion and migration ability of pancreatic cancer cells, transwell assays were conducted and results showed that the invasion and migration ability of PANC-1 cells and SW1990 cells was significantly reduced after specific knockdown of Numb PRR^L^ (Fig. 4B). These results indicate that Numb PRR^L^ plays a major role in maintaining the malignant biological behavior of pancreatic cancer. Furthermore, Numb PRR^L^ can affect EMT in pancreatic cancer cells. After Numb PRR^L^ specific knockdown, the expression of E-cadherin was up-regulated, while the expression of N-cadherin, Vimentin, Fibronectin, Snail2 and ZEB1 were down-regulated (Fig. 4C), suggesting that Numb PRR^L^ can promote EMT in pancreatic cancer cells.

### ATP11A regulated the expression of ZEB1 and Snail2 by regulating Numb PRR^L^, thus affecting EMT

**Figure 5:**
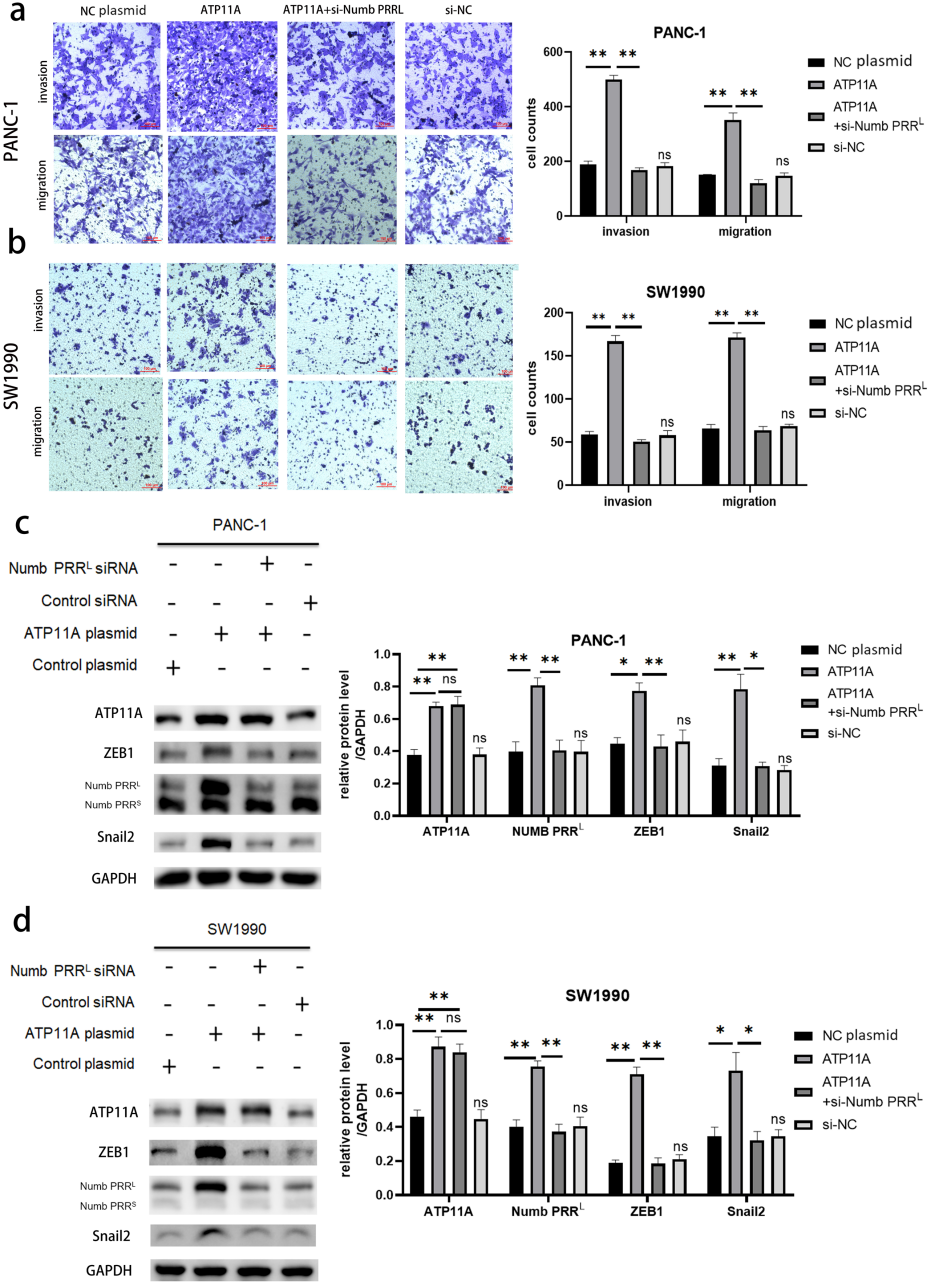
Rescue experiments. ATP11A affects the expression of ZEB1/Snail2 and the invasion and migration ability of pancreatic cancer cells by regulating Numb PRR^L^. (A, B) The effect of ATP11A in the invasion and migration ability of PANC-1 (A) and SW1990 (B) cells was blocked by specific knockdown of Numb PRR^L^. (C, D) The promoter effect of ATP11A on the expression of ZEB1 and Snail2 in PANC-1 (C) cell lines and SW1990 (D) cell lines was blocked by specific knockdown of Numb PRR^L^. Each experiment was repeated three times. The differences of WB analysis, cell migration and invasion assays were expressed as mean ± S.E. and compared by Student’s *t*-test. *P < 0.05; **P < 0.01; ns, no significance.

Rescue experiments were performed in order to explore the association between ATP11A, Numb PRR^L^, and transcription factors ZEB1/Snail2. Numb PRR^L^ was specifically knocked down after the overexpression of ATP11A, then observing whether the effect of ATP11A overexpression on the biological behavior of pancreatic cancer cells was recovered. Results indicated that the increased invasion and migration ability induced by ATP11A was blocked after specific inhibition of Numb PRR^L^ (Figs. 5A, 5B ). In addition, western blot assay results showed that specific knockdown of Numb PRR^L^ did not affect the expression of ATP11A, but the up-regulation of ZEB1 and Snail2 induced by ATP11A was blocked (Figs. 5C, 5D). These results suggest that ATP11A up-regulates the expression of ZEB1 and Snail2 by regulating Numb PRR^L^.

### ATP11A coimmunoprecipitates with Numb PRR^L^ directly in PC cells

**Figure 6:**
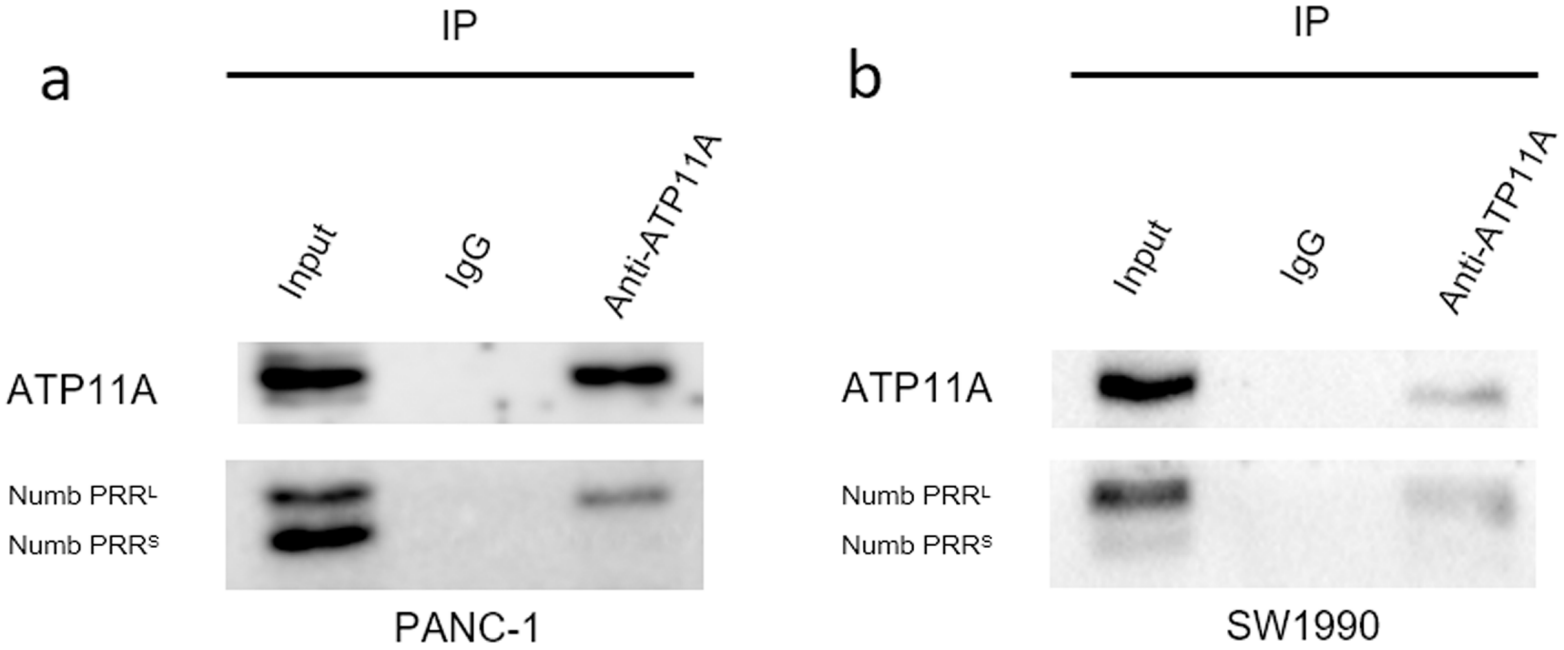
The close interaction between Numb and ATP11A in PC cell lines. PANC-1 (A) and SW1990 (B) lysates were immunoprecipitated and subjected to a western blot analysis. Input and IgG lanes show the positive and negative controls, respectively.

Immunoprecipitation assays can detect direct binding of two proteins in cells. Our immunoprecipitation results (Fig. 6) showed that ATP11A could specifically bind to Numb PRR^L^, but could hardly bind to Numb PRR^S^ in PANC-1 and SW1990 cells. These results indicate that ATP11A and the Numb PRR^L^ protein have a direct binding effect in pancreatic cancer cells.

### The effect of ATP11A on EMT is TGFB dependent

**Figure 7:**
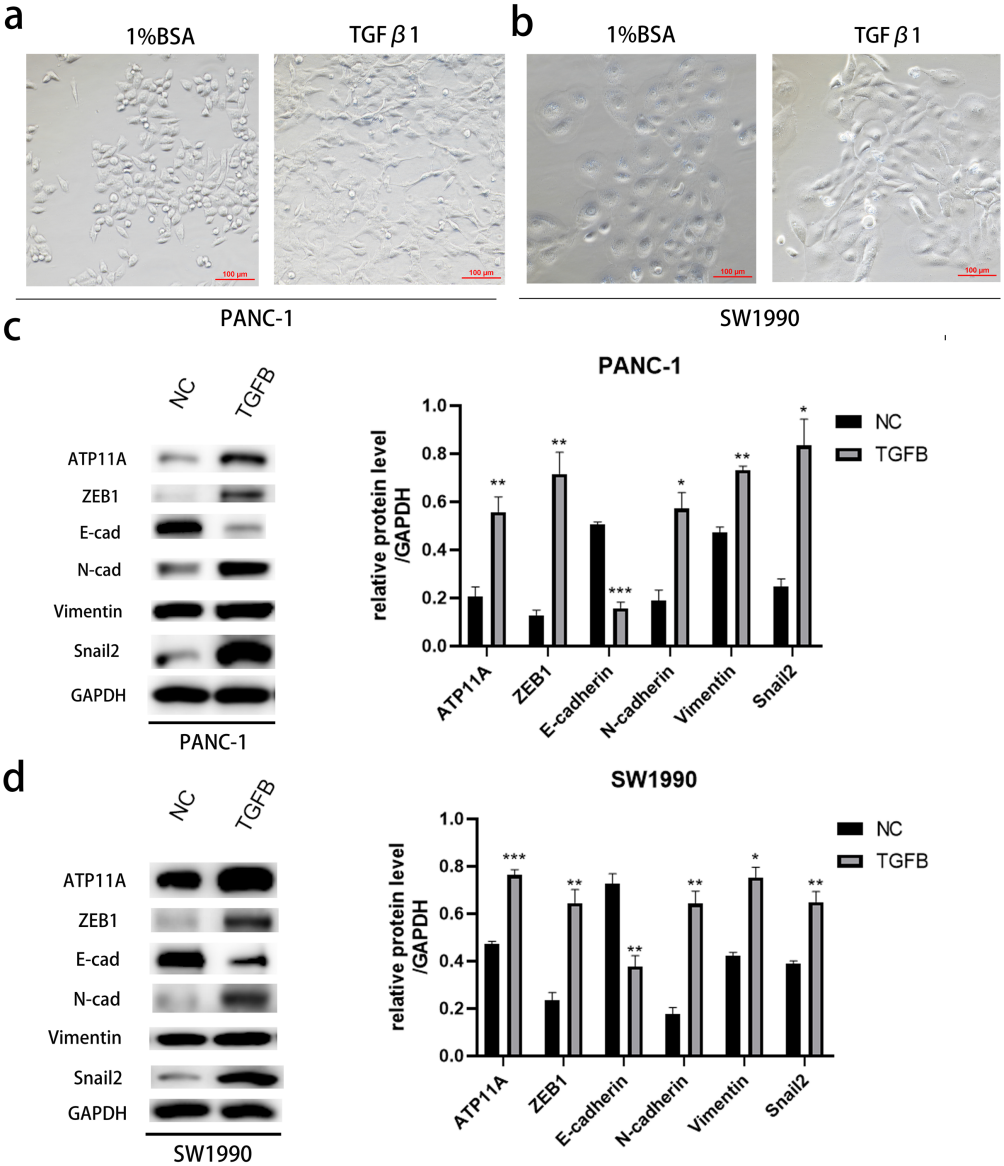
The promoting effect of ATP11A on EMT is TGFB dependent. (A, B) EMT-like changes were observed in the cell morphology of PANC-1 (A) and SW1990 (B) 48 h after TGFβ1 treatment (10 ng/ml). (C, D) TGFB promotes the expression of ATP11A and EMT-related proteins. (C) Changes in ATP11A and EMT-related proteins after TGFβ1 treatment in PANC-1 cells. (D) Changes in ATP11A and EMT-related proteins after TGFβ1 treatment in SW1990 cells. Each experiment was repeated three times. The differences of WB analysis were expressed as mean ± SEM and compared by Student’s *t*-test. *P < 0.05; **P < 0.01; ***P < 0.001; ns, no significance.

After induction with TGFB1, PANC-1 and SW1990, cells showed obvious morphological changes (Figs. 7A, 7B). To explore whether TGFB can promote the expression of ATP11A, cell proteins were extracted and WB experiments were conducted. The results showed that after TGFB induction, the expression of ATP11A significantly increased, accompanied by changes of EMT-related proteins: the expression of E-Cadherin was down-regulated, and the expression of N-Cadherin, Vimentin, Fibronectin, Snail2 and ZEB1 were up-regulated (Figs. 7C, 7D). This indicates that the promoting effect of ATP11A on EMT is TGFB dependent.

## Discussion

ATP11A is widely expressed in mammals (Segawa, Kurata & Nagata, 2016). A previous study reported that ATP11A is a predictive prognosis marker of colorectal cancer (CRC) (Miyoshi et al., 2009) because its mRNA level was significantly higher in CRC tissues compared to normal tissues. In this study, the mRNA level of *ATP11A* was significantly higher in pancreatic cancer tissues than in normal tissues, and there was a strong correlation between the mRNA level of ATP11A and Numb. Western blot and immunohistochemical results confirmed that the ATP11A protein level was significantly higher in pancreatic cancer tissues than in adjacent tissues, and the ATP11A protein level was correlated with tumor stage. In addition, patients with high ATP11A expression had a worse prognosis. Transwell assay results showed that increased ATP11A expression can increase the invasion and migration ability of pancreatic cancer cells, and *vice versa*.

Numb is considered to be a key gene which determines the fate of *Drosophilus sativum* (Uemura et al., 1989). Moreover, studies have shown that Numb plays critical roles in mammalian neural development (Cayouette & Raff, 2002; Verdi et al., 1996), and is closely associated with the development and progression of multiple cancers (Pece et al., 2004; Westhoff et al., 2009). Accumulating evidence has proposed that Numb is a tumor suppressor (Pece et al., 2011; Sheng et al., 2017). A previous study reported that the alternative splicing of Numb transcripts leads to two main isoforms that vary in the lengths of their proline-rich regions (PRR) length (Yan, 2010). Numb PRR^L^ advanced proliferation yet did not affect differentiation, while Numb PRR^S^ advanced differentiation but did not affect proliferation in murine embryonic carcinoma cells (Karaczyn et al., 2010; Verdi et al., 1999). The anti-tumor effect of Numb is becoming increasingly apparent as a result of the rapid development of related research. Studies conducted on non-small-cell lung cancer (Misquitta-Ali et al., 2011; Zong et al., 2014), invasive bladder cancer (Zhang et al., 2014), and liver cancer (Lu et al., 2015) have shown that the Numb PRR^L^ expression in tumors was higher than in normal tissues. The expression level of Numb PRR^L^ was also correlated with an unfavourable prognosis. In pancreatic cancer tissues, Numb PRR^L^ has a higher expression than in normal tissues (Zheng et al., 2015).

In our study, the relationship between ATP11A and Numb was identified. Our results showed that the overexpression or knockdown of ATP11A led to a corresponding increase or decrease of Numb PRR^L^. Similar results in two types of pancreatic cancer cells were obtained, suggesting that ATP11A may influence the biological behavior of tumours in pancreatic carcinoma by regulating Numb PRR^L^.

Moreover, we found that ATP11A can promote the epithelial-to-mesenchymal transition (EMT) of pancreatic cancer. Our results indicated that ATP11A can upregulate the expression of ZEB1, which is a prime component of a network of transcription factors that control EMT (Caramel, Ligier & Puisieux, 2018) and appears to be a central switch that decides cell fate (Caramel, Ligier & Puisieux, 2018). In pancreatic cancer, ZEB1 has been identified as a critical factor of cell plasticity, and one that advances metastasis (Krebs et al., 2017). In addition to ZEB1, ATP11A also can promote the expression of Snail2. Snail2 is an important transcription factor in EMT, and plays an important role in inhibiting the expression of E-cadherin (Hajra, Chen & Fearon, 2002). Snail2 is also an essential mediator of Twist1-induced epithelial mesenchymal transition and metastasis (Casas et al., 2011). Numb also has a regulatory effect on EMT (Karaczyn et al., 2017; Wang & Li, 2010). Previous studies have reported that Numb appears to assume a significant part of the proper functions of the Par protein complex (Nishimura & Kaibuchi, 2007) and in cell-cell junctions (Rasin et al., 2007), both of which are associated with EMT.

Interestingly, we found that ATP11A can promote the expression of SRPK2, which is thought to reduce the expression of Numb PRR^S^ in HCC (Lu et al., 2015). However, no changes in the expression of Numb PRR^S^ were observed in our study, indicating that the regulation of Numb in pancreatic cancer is different from that in other cancers. Whether SRPK2 affects the expression of Numb in pancreatic cancer may be worth studying in the future.

Subsequently, the expression of Numb PRR^L^ was detected in pancreatic and paracancer tissues, which indicated that Numb PRR^L^ was highly expressed in pancreatic cancer tissues. Further, we found that Numb PRR^L^ plays an important role in maintaining the invasion and migration of pancreatic cancer cells, and promotes the EMT of pancreatic cancer cells. Furthermore, rescue experiments were conducted to determine whether ATP11A can regulate the expression of ZEB1 and Snail2 by affecting Numb PRR^L^. Our results indicated that the promotion effect of ATP11A on ZEB1/Snail2 was successfully inhibited after Numb PRR^L^ was specifically knocked down. Meanwhile, the increased invasion and migration ability in tumor cells caused by the over expression of ATP11A was also inhibited, indicating that ATP11A regulated the expression of ZEB1 and Snail2 by affecting Numb PRR^L^, ultimately affecting the invasion and migration ability of pancreatic carcinoma cells.

Nilsson, Jonsson & Tuck (2011) reported that the Numb of *Caenorhabditis elegans* binds TAT-1 aminophospholipid translocase, a P4 family adenosine triphosphatase. ATP11A also serves as a catalytic part of the P4-ATPase-inverting enzyme complex in mammals. Therefore, we speculate that ATP11A also binds Numb. Immunoprecipitation experiments were also conducted in this study and found that ATP11A can specifically bind Numb PRR^L^, which further confirms our results.

Finally, TGFB is a major driver of EMT in pancreatic cancer (Su et al., 2020; Xu, Lamouille & Derynck, 2009). We wanted to know whether the effect of ATP11A on EMT is regulated by TGFB. After TGFB stimulation, significant up-regulation of ATP11A expression was detected, accompanied by changes in EMT-related proteins, suggesting that ATP11A is TGFB dependent in promoting EMT. Our study expands the mechanism of TGFB-induced epithelial to mesenchymal transition, and shows a potential pathway that could be used therapeutically to inhibit EMT.

## Conclusion

In summary, ATP11A is highly expressed in pancreatic carcinoma and is associated with adverse prognosis. Furthermore, ATP11A affects the epithelial-to-mesenchymal transition (EMT) of pancreatic cancer by regulating the TGFB dependent Numb PRR^L^-ZEB1/Snail2 pathway.

## Supplemental Information

10.7717/peerj.13172/supp-1 Supplemental Information 1 Raw data of western blot.

10.7717/peerj.13172/supp-2 Supplemental Information 2 Raw data.

10.7717/peerj.13172/supp-3 Supplemental Information 3 Sequences of ATP11A plasmids.

## Additional Information and Declarations

### Data Availability

The following information was supplied regarding data availability: The raw data is available in the Supplemental Files.
