# Supplementary material for: ATP11A promotes EMT by regulating Numb PRRL in pancreatic cancer cells
Source: PeerJ. 2022 Mar 23;10:e13172. doi: 10.7717/peerj.13172 (PMC8957272; doi:10.7717/peerj.13172)
Supplement: Supplemental Information 3 [file peerj-10-13172-s003.pdf]

Customer order number:GOSE0222462

Overexpression carrier build information sheet

Gike gene

One Overexpression carrier construction

Genetic information

Genetic name:ATP11A (NM_015205).

Species:Human

1. Carrier enzyme cut

1.1 Carrier information:

Carrier name:GV230

Component order:CMV-MCS-EGFP-SV40-Neomycin

Clone site:XhoI / KpnI

Control number:CON083

Carrier map:

http://www.genechem.com.cn/service/index.php?ac=gene&at=vector_search&keyword=GV230 The carrier instructions are available for download

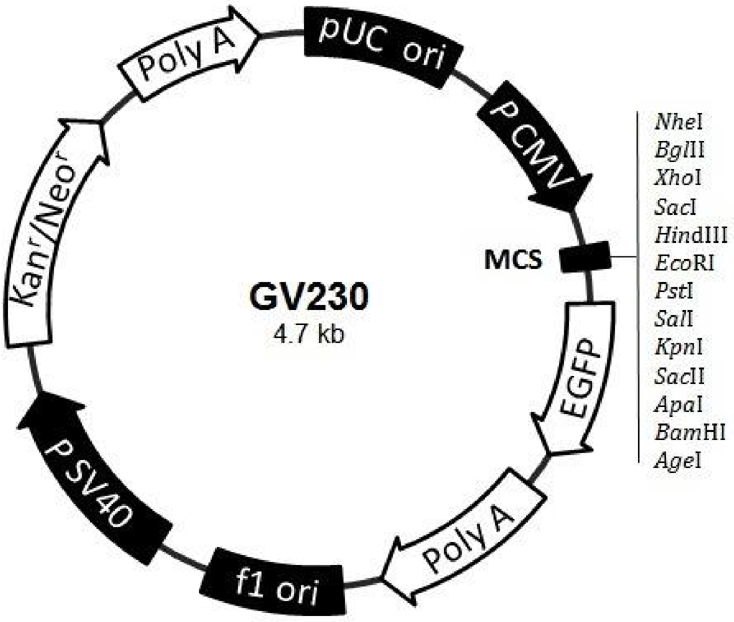

1.2 Enzyme cut results:

Electrophoresis:

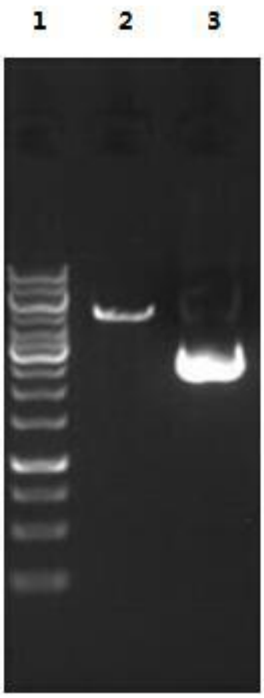

Electrophoresis diagram illustration:

1#: 10kb Marker (From top to bottom, the strips are:10kb、8kb、6kb、5kb、4kb、3.5kb、3kb、2.5kb、2kb、

1.5kb、1kb、750bp、500bp、250bp)

2: Carrier enzyme cut product

Unenzyme-cut vectors (plasmids extracted from bacteria have different migrations due to the presence of superspirules, open rings, linear and other conformations.)

Rate, in agar gelling electrophoresis presents different size stripes, so at this time the plasmid electrophoresis band can only be judged as the size of plasmid molecular weight

reference, can not be used as an accurate basis for judgment. After digestion by single enzyme cutting, the plasmids present a homogeneous electrophoretic band, which can be paired with Marker

than to determine its molecular weight size)

Note: Due to the company's quality control needs, the carrier after a large number of enzyme cutting, after quality identification, quality qualified enzyme cutting fragments will be a large number of preservation for use

The carrier is built for multiple users, so the picture appears in multiple users' reports, so customers should use the diagram carefully. If necessary to publish an article

To, please mark in the experimental material:GV230 carrier,XhoI / KpnI enzyme cutting, purchased from Shanghai Jikai Gene Chemistry Technology Co., Ltd.

2. Acquisition of the target gene fragment

2.1 Primer

ID	seq
ATP11A(57190-1)-p1	TACCGGACTCAGATCTCGAGCGCCACCATGGACTGCAGCCTCGTGCGGACGCTC
ATP11A(57190-1)-p2	GATCCCGGGCCCGCGGTACCGTGAAACTCAGGCTGCTGGAAGTCTGAGAAAG

Primer Description: Contains exchange pairing base, enzyme cutting point, and contains the target gene 5' end part of the sequence for PCR fishing purpose gene

2.2 PCR results

PCR product size:3451

Electrophoresis:

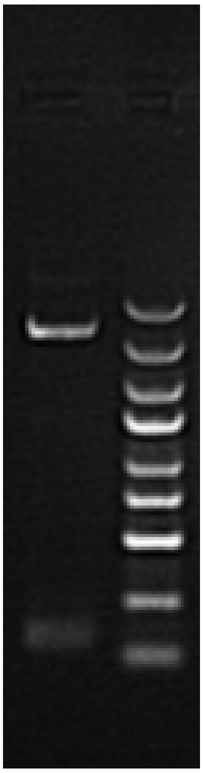

Electrophoresis diagram illustration:

Marker is top-down:5 kb,3 kb,2 kb,1.5 kb,1 Kb,750 bp,500bp,250 bp,100 bp

3. Recombinant plasmid build

3.1 The product is exchanged into a linear expression carrier

3.2 PCR identification primer

ID	seq
KL57190-p3	CTGCCGGAGCTGCAGCGCGG
KL57190-p4	CGTCGCCGTCCAGCTCGACCAG

3.3 PCR identification recombinant clones

Positive converter PCR product size:1145

Electrophoresis:

1 2 3 4 5 6 7 8 9 10 11 12

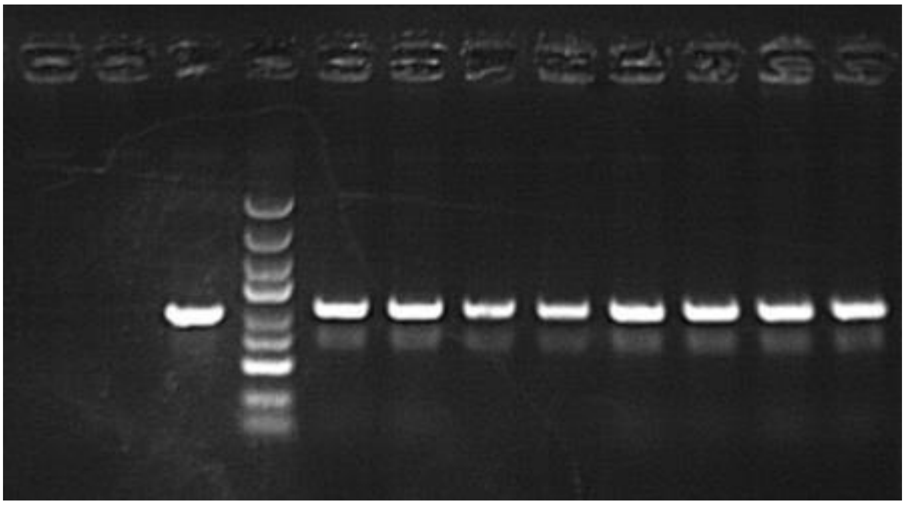

Electrophoresis diagram illustration:

1: Negative control(ddH2O).

2:Negative control (empty from the control group).

3: Positive control(GAPDH).

4:Marker is 5 kb, 3 kb,2 kb,1.5 kb,1 Kb,750 bp, 500bp,250 bp,100 bp fromtop to bottom

5-12:1-8 converter

4. Analysis of positive cloning sequencing results and results:

Match results:

GCTACCGGACTCAGATCTCGAGCGCCACCATGGACTGCAGCCTCGTGCGGACGCTCGTGCACAGATACTGTG
CAGGAGAAGAGAATTGGGTGGACAGCAGGACCATCTACGTGGGACACAGGGAGCCACCTCCGGGCGCAGAGG
CCTACATCCCACAGAGATACCCAGACAACAGGATCGTCTCGTCCAAGTACACATTTTGGAACTTTATACCCA
AGAATTTATTTGAACAATTCAGAAGAGTAGCCAACTTTTATTTCCTTATCATATTTCTGGTGCAGTTGATTA
TTGATACACCCACAAGTCCAGTGACAAGCGGACTTCCACTCTTCTTTGTCATTACTGTGACGGCTATCAAAC
AGGGTTATGAAGACTGGCTTCGACATAAAGCAGACAATGCCATGAACCAGTGTCCTGTTCATTTCATTCAGC
ACGGCAAGCTCGTTCGGAAACAAAGTCGAAAGCTGCGAGTTGGGGACATTGTCATGGTTAAGGAGGACGAG
ACCTTTCCCTGCGACTTGATCTTCCTTTCCAGCAACCGGGGAGATGGGACGTGCCACGTCACCACCGCCAGC
TTGGATGGAGAATCCAGCCATAAAACGCATTACGCGGTCCAGGACACCAAAGGCTTCCACACAGAGGAGGAT
ATCGGCGGACTTCACGCCACCATCGAGTGTGAGCAGCCCCAGCCCGACCTCTACAAGTTCGTGGGTCGCATCA
ACGTTTACAGTGACCTGAATGACCCCGTGGTGAGGCCCTTAGGATCGGAAAACCTGCTGCTTAGAGGAGCTA
CACTGAAGAACACTGAGAAAATCTTTGGTGTGGCTATTTACACGGGAATGGAAACCAAGATGGCATTAAAT
TATCAATCAAAATCTCAGAAGCGATCTGCCGTGGAAAAATCGATGAATGCGTTCCTCATTGTGTATCTCTGC
ATTCTGATCAGCAAAGCCCTGATAAACACTGTGCTGAAATACATGTGGCAGAGTGAGCCCTTTCGGGATGAG
CCGTGGTATAATCAGAAAACGGAGTCGGAAAGGCAGAGGAATCTGTTCCTCAAGGCATTCACGGACTTCCTG
GCCTTCATGGTCCTCTTTAACTACATCATCCCTGTGTCCATGTACGTCACGGTCGAGATGCAGAAGTTCCTC
GGCTCTTACTTCATCACCTGGGACGAAGACATGTTTGACGAGGAGACTGGCGAGGGGCCTCTGGTGAACACG
TCGGACCTCAATGAAGAGCTGGGACAGGTGGAGTACATCTTCACAGACAAGACCGGCACCCTCACGGAAAAC
AACATGGAGTTCAAGGAGTGCTGCATCGAAGGCCATGTCTACGTGCCC
CACGTCATCTGCAACGGGCAGGTCCTCCCAGAGTCGTCAGGAATCGACATGATTGACTCGTCCCCCAGCGTC
AACGGGAGGGAGCGCGAGGAGCTGTTTTTCCGGGCCCTCTGTCTCTGCCACACCGTCCAGGTGAAAGACGAT
GACAGCGTAGACGGCCCCAGGAAATCGCCGGACGGGGGGAAATCCTGTGTGTACATCTCATCCTCGCCCGAC
GAGGTGGCGCTGGTCGAAGGTGTCCAGAGACTTGGCTTTACCTACCTAAGGCTGAAGGACAATTACATGGAG
ATATTAAACAGGGAGAACCACATCGAAAGGTTTGAATTGCTGGAAATTTTGAGTTTTGACTCAGTCAGAAG
GAGAATGAGTGTAATTGTAAAATCTGCTACAGGAGAAATTTATCTGTTTTGCAAAGGAGCAGATTCTTCGA
TATTCCCCCGAGTGATAGAAGGCAAAGTTGACCAGATCCGAGCCAGAGTGGAGCGTAACGCAGTGGAGGGGC
TCCGAACTTTGTGTGTTGCTTATAAAAGGCTGATCCAAGAAGAATATGAAGGCATTTGTAAGCTGCTGCAG
GCTGCCAAAGTGGCCCTTCAAGATCGAGAGAAAAAGTTAGCAGAAGCCTATGAGCAAATAGAGAAAGATCT
TACTCTGCTTGGTGCTACAGCTGTTGAGGACCGGCTGCAGGAGAAAGCTGCAGACACCATCGAGGCCCTGCA
GAAGGCCGGGATCAAAGTCTGGGTTCTCACGGGAGACAAGATGGAGACGGCC
GCGGCCACGTGCTACGCCTGCAAGCTCTTCCGCAGGAACACGCAGCTGCTGGAGCTGACCACCAAGAGGATC
GAGGAGCAGAGCCTGCACGACGTCCTGTTCGAGCTGAGCAAGACGGTCCTGCGCCACAGCGGGAGCCTGACC
AGAGACAACCTGTCCGGACTTTCAGCAGATATGCAGGACTACGGTTTAATTATCGACGGAGCTGCACTGTCT
CTGATAATGAAGCCTCGAGAAGACGGGAGTTCCGGCAACTACAGGGAGCTCTTCCTGGAAATCTGCCGGAGC
TGCAGCGCGGTGCTCTGCTGCCGCATGGCGCCCTTGCAGAAGGCTCAGATTGTTAAATTAATCAAATTTTCA
AAAGAGCACCCAATCACGTTAGCAATTGGCGATGGTGCAAATGATGTCAGCATGATTCTGGAAGCGCACGTG
GGCATAGGTGTCATCGGCAAGGAAGGCCGCCAGGCTGCCAGGAACAGCGACTATGCAATCCCAAAGTTTAAG
CATTTGAAGAAGATGCTGCTTGTTCACGGGCATTTTTATTACATTAGGATCTCTGAGCTCGTGCAGTACTTC
TTCTATAAGAACGTCTGCTTCATCTTCCCTCAGTTTTTATACCAGTTCTTCTGTGGGTTTTCACAACAGACT
TTGTACGACACCGCGTATCTGACCCTCTACAAC

ATCAGCTTCACCTCTCTCCCCATCCTCTCCTGTACAGCCTCATTCATGGAGCAGCATGGGGGGGGGCATGACG
GGGGGAGACCCCGGGGGGGGGGGGGGTCTTTGGTGCTTATTTCGTTTGAGAATACAACTGGAGACACACAC
AACGGGGGGAGATTGGGGACTGGGGGTTTGGAGACCGCTGGATTCACCGTGGGTGGTCACAGTTACACTAA
AGCTTGCATTGGACACTACTGGACTACTTGGCACACACCATTTTGTCATCTGGGGGGTCGCTGCTGTTCTAC
GTTTTTTTTTTTGCTTCTGGGGGGGGGGGGGGATCTGGGGGTTCACCCACACAGAGAGTACTACGTTTTCAT
CCAGATGGTTCWGGGGGCCTGGGGCCATCGTGCTGGGGTGACCACCTCTCTCTCCCCCCCTCCTCAAGAAAG
TCCTGTTGGGGGCAGCTGTGGCCAACAGCAAGAGAGAGGTCCAGACTAAGAGCCAGTGCCTTTGGGCAGTCA
CACATCTTTATGCTTTCTCTCAGACTTCCAGCACTGAGTTTC
ANDGGTACCGCGGGCCCGGGGGCACCGGGGTCGCCACCATCATGGGAGA

Compare the results to show that the ok is measured
